# Application of matrix-assisted laser desorption/ionization mass spectrometry to identify species of Neotropical *Anopheles* vectors of malaria

**DOI:** 10.1186/s12936-019-2723-0

**Published:** 2019-03-22

**Authors:** Jose R. Loaiza, Alejandro Almanza, Juan C. Rojas, Luis Mejía, Norma D. Cervantes, Javier E. Sanchez-Galan, Fernando Merchán, Arnaud Grillet, Matthew J. Miller, Luis F. De León, Rolando A. Gittens

**Affiliations:** 1 Centro de Biodiversidad y Descubrimiento de Drogas, Instituto de Investigaciones Científicas y Servicios de Alta Tecnología (INDICASAT AIP), City of Knowledge, Panama, 0843-01103 Republic of Panama; 2 Smithsonian Tropical Research Institute, Panama, Republic of Panama; 3 Programa Centroamericano de Maestría en Entomología, Universidad de Panamá, Panama, Republic of Panama; 4 College of Health Sciences, The University of Texas at El Paso, El Paso, TX USA; 5 Grupo de Investigación en Biotecnología, Bioinformática y Biología de Sistemas, Centro de Producción e Investigaciones Agroindustriales, Universidad Tecnológica de Panamá, Panama, Republic of Panama; 6 Grupo de Investigación en Sistemas de Comunicaciones Digitales Avanzados, Facultad de Ingeniería Eléctrica, Universidad Tecnológica de Panamá, Panama, Republic of Panama; 7 ENSEIRB-MATMECA-Bordeaux INP, Talence, France; 8 Sam Noble Oklahoma Museum of Natural History and Department of Biology, University of Oklahoma, Norman, OK USA; 9 Department of Biology, University of Massachusetts Boston, Boston, MA USA; 10 Centro de Neurociencias, INDICASAT AIP, Panama, Republic of Panama

**Keywords:** *Anopheles* mosquito, Taxonomic identification, MALDI, Mass spectrometry, Malaria vector, Panama

## Abstract

**Background:**

Malaria control in Panama is problematic due to the high diversity of morphologically similar *Anopheles* mosquito species, which makes identification of vectors of human *Plasmodium* challenging. Strategies by Panamanian health authorities to bring malaria under control targeting *Anopheles* vectors could be ineffective if they tackle a misidentified species.

**Methods:**

A rapid mass spectrometry identification procedure was developed to accurately and timely sort out field-collected Neotropical *Anopheles* mosquitoes into vector and non-vector species. Matrix-assisted laser desorption/ionization (MALDI) mass spectra of highly-abundant proteins were generated from laboratory-reared mosquitoes using different extraction protocols, body parts, and sexes to minimize the amount of material from specimen vouchers needed and optimize the protocol for taxonomic identification. Subsequently, the mass spectra of field-collected Neotropical *Anopheles* mosquito species were classified using a combination of custom-made unsupervised (i.e., Principal component analysis—PCA) and supervised (i.e., Linear discriminant analysis—LDA) classification algorithms.

**Results:**

Regardless of the protocol used or the mosquito species and sex, the legs contained the least intra-specific variability with enough well-preserved proteins to differentiate among distinct biological species, consistent with previous literature. After minimizing the amount of material needed from the voucher, one leg was enough to produce reliable spectra between specimens. Further, both PCA and LDA were able to classify up to 12 mosquito species, from different subgenera and seven geographically spread localities across Panama using mass spectra from one leg pair. LDA demonstrated high discriminatory power and consistency, with validation and cross-validation positive identification rates above 93% at the species level.

**Conclusion:**

The selected sample processing procedure can be used to identify field-collected *Anopheles* species, including vectors of *Plasmodium*, in a short period of time, with a minimal amount of tissue and without the need of an expert mosquito taxonomist. This strategy to analyse protein spectra overcomes the drawbacks of working without a reference library to classify unknown samples. Finally, this MALDI approach can aid ongoing malaria eradication efforts in Panama and other countries with large number of mosquito’s species by improving vector surveillance in epidemic-prone sites such as indigenous *Comarcas*.

**Electronic supplementary material:**

The online version of this article (10.1186/s12936-019-2723-0) contains supplementary material, which is available to authorized users.

## Background

Despite historical and ongoing eradication efforts, human malaria transmitted by *Anopheles* mosquitoes continues to be a major public health concern around the world [1]. Malaria was one of the leading causes of death during the construction of the Panamanian Interoceanic Canal in the early 1900s. In Panama, malaria prevalence oscillated dramatically during the last 50 years, with sporadic and/or cyclical epidemics every five to 10 years [2–4]. Recently however, from 2001 to 2005, a malaria outbreak was documented in indigenous territories known as “*Comarcas*” where a sixfold increase in the number of cases was observed [5, 6]. This epidemic was controlled during subsequent years, and the number of symptomatic cases in the country has dropped considerably since this event. Nonetheless, malaria is still endemic in Panama, and there is potential for future outbreaks particularly in indigenous *Comarcas* with health, social and demographic disparities [3, 6].

Malaria control in Panama is done mainly through eradication of mosquito vectors using toxic insecticides. This strategy requires that the *Anopheles* species responsible for transmission be promptly and accurately identified. Nonetheless, identification in Panama is problematic due to a high number of morphologically similar *Anopheles* species [7]. Control strategies to bring malaria down targeting *Anopheles* vectors could be ineffective if they tackle a misidentified non-vector species. This is likely the case in Panamanian indigenous *Comarcas* where as many as 10 *Anopheles* species occur in a single locality, and where 40% of these are expected to transmit both *Plasmodium vivax* and *P. falciparum* to humans [5, 8]. The identification of *Anopheles* mosquitoes in Panama is done using traditional morphological approaches (e.g., dichotomic keys), but this approach requires meticulous taxonomic training and a great deal of entomological expertise [9, 10]. Also, it is time consuming and could be impractical when inspecting numerous samples [11]. Hence, Panamanian health authorities require new approaches to accurately and timely sort out vector from non-vector *Anopheles* species.

DNA barcoding is a valid alternative to identify arthropod species because it has better taxonomic resolution than morphological approaches, even if the promise of being less expensive has not yet materialized [12]. DNA barcodes work well with a small amount of tissue, and do not require prior knowledge of insect morphology [13]. However, generating DNA barcodes requires advanced sample preparation and proper laboratory facilities to extract, amplify and sequence nucleic acids, most of which are rarely found in developing countries where arthropod-borne infections like malaria prevail [14].

In recent years, matrix–assisted laser desorption/ionization (MALDI) mass spectrometry has become an alternative for arthropod taxonomic identification [10, 15, 16]. This method has been used effectively to study several aspects of vector biology, including taxonomic status (i.e., species boundaries), pathogen infection rates and food source identity [17–21]. MALDI mass spectrometry uses a profile of the most abundant proteins to “fingerprint” biological samples, and thus, is conceptually similar to DNA barcoding, but possibly cheaper on a per sample basis. Furthermore, MALDI can generate accurate identifications in just a few hours, rather than 5 to 10 days as in the case of DNA barcoding even in “rush” cases [22]. Previous efforts with MALDI to taxonomically classify members of family Culicidae were successful using both laboratory-reared and field-collected specimens, and specific body parts (e.g., thorax, cephalothorax and/or legs) plus samples from different regions of the world [9, 10, 23–25]. Yssouf et al. [9, 10] used MALDI with all six legs to classify mosquito species from Africa, Europe and the US while Mewara et al. [24], using the same approach, accurately identified specimens of four different mosquito genera in Northern India. More recently in France, Vega-Rúa et al. [25] designed a double entry query protocol with MALDI protein spectra obtained from thoraxes and legs to improve the identification of morphologically compromised specimens. Hence, MALDI’s accurate and rapid identification capabilities might prove ideal to solve the shortcomings of taxonomically classifying *Anopheles* mosquitoes in Panama, thus assisting ongoing malaria eradication efforts by improving the vector surveillance system in indigenous *Comarcas*.

Here, a methodology based on previously published extraction protocols was adjusted and assessed the accuracy of MALDI identification with a small portion of tissue from the mosquito body to otherwise preserve a specimen voucher. Different statistical procedures were also explored to analyse and classify protein spectra from field-collected mosquitoes, which are difficult to evaluate with currently available strategies from commercial vendors, including working without a reference library of well curated protein spectra. Specifically, the authors ask if MALDI mass spectrometry can discriminate among field-collected individuals of 11 known mosquito species in the genus *Anopheles*, including taxa that are vectors of human *Plasmodium* in Panama, plus *Chagasia bathana*, a closely phylogenetically related and ancestral species to *Anopheles*.

## Methods

### Sample preparation and optimization

Initial experiments were conducted with laboratory-reared mosquitoes from three discrete biological species: *Anopheles albimanus* (vector of malaria); *Aedes aegypti* (vector of Zika and dengue); and *Aedes albopictus* (vector of Chikungunya) (Additional file 1). Three different sample preparation protocols (i.e., protein extraction methods), adapted with minor modifications from previous studies [10, 20], were compared and the one with the most suitable results got selected for further experimentation with field-collected specimens (see the full description of these protocols in Table 1). To test for differences in the mass spectra produced with the three extraction protocols, whole insect-bodies of freshly emerged and starved female mosquitoes were used to avoid noise in the acquired protein signal. Two hundred and twenty-five female mosquitoes were used in total at this point, 25 individuals per species for each protocol. Further, different parts of the body of female mosquitoes (e.g., head, thorax, abdomen, wings and one of the anterior, middle and posterior legs) were assessed to confirm if they contained different protein spectra, and if these spectra were consistent across specimens of the same taxon as it has been shown previously [9, 10, 23–25]. Body parts were dissected using a micro-dissecting kit, placed in separate micro-centrifuge tubes and labeled accordingly. For this evaluation, another 25 lab-reared female individuals of *A. albimanus*, *A. aegypti* and *A. albopictus* (Additional files 2 and 3) were used. Finally, the section of the body with the highest and most consistent protein signal was selected and proceeded to compare whether or not females and males of a given species display differences in their protein spectra as shown by previous studies using whole insect bodies [20]. Once more, differences between females and males were evaluated using 25 laboratory-reared specimens of *A. albimanus*, *A. aegypti* and *A. albopictus*, respectively (Additional file 4).

**Table 1:** Description of three different MALDI mass spectrometry protein-extraction protocols used in the present study

Protocol #1	Protocol #2	Protocol #3
Selected mosquito body parts were placed in separate microcentrifuge tubes, rinsed with 300 μL ultrapure water and 900 μL ethanol, and centrifuged at 13,000 rpm for 2 min Samples were decanted and treated with 10 μL of 70% formic acid for 5 min at room temperature Immediately after, samples were homogenized in the tube with the help of a manual pestle with an additional 10 μL of 100% acetonitrile and centrifuged at 13,000 rpm for 2 min A small volume of supernatant was pre-mixed with equal volume of 10 mg/mL α-cyano-4-hydroxycinnamic acid (HCCA) matrix and 1 μL of the mix was quickly placed in its respective target well in triplicate	Selected mosquito body parts were rinsed with distilled water and dried with paper Samples were immediately homogenized with the help of a manual pestle in 20 μL of 70% formic acid and 20 μL of 100% acetonitrile and incubated for 1 h Samples were vortexed for 15 s, centrifuged at 13,000 rpm for 2 min and a small volume of the supernatant was pre-mixed with equal volume of 10 mg/mL HCCA before adding 1 μL of the mix it to the target well in triplicate	Selected mosquito body parts were homogenized with the help of a manual pestle in 20 μL of 10% formic acid, pre-mixing with 1.5 × volume of sinapinic acid matrix, and centrifuged at 13,000 rpm for 2 min 1 μL of supernatant was immediately added to its respective target well in triplicate

Protocol #1: Formic acid/ethanol extraction protocol recommended by the MALDI manufacturer for bacterial identification (Bruker, Bremen, Germany)

Protocol #2: Protocol based on the method proposed by Yssouf et al. [9], with minor modifications

Protocol #3: Protocol based on the method proposed by Müller et al. [22], with minor modifications

### Field-collected Neotropical *Anopheles* species

For the second part of the study, fresh *Anopheles* mosquitoes from four subgenera and seven geographically spread localities in indigenous *Comarcas* across Panama (Table 2) were collected. Mosquitoes were collected at night during seven consecutive days per location, using different types of traps (e.g., Human Landing Catch, Intersection, Shannon and Center for Disease Control—CDC—miniature light trap) (Additional file 5). Samples were stored at room temperature in individual, dry microtubes along with silica gel, and transported back to the laboratory in plastic bags. Once in the laboratory, mosquitoes were maintained at − 20 °C to preserve the integrity of their proteins. Initially, all field-collected specimens were sorted and identified to species level using a taxonomic key based on morphological characters of the female [26]. Then, between ten and 66 individuals per species were processed and analysed using mass spectrometry, for a total of 12 species and 299 specimens (Table 2). For this section of the study, and upon analysing the outcomes of experiments performed during the first part of the methodology, the best extraction protocol and the section of the mosquito body and sex with the highest protein signal and consistency were used. The goal here was to determine if different Neotropical *Anopheles* species, non-vectors and vectors of human *Plasmodium*, had specific protein profiles generated with MALDI that could be used for rapid and accurate identification purposes.

**Table 2:** Description of samples subjected to analysis with the MALDI mass spectrometry procedure

Mosquito species	# of specimens	Locality code	# of expected spectra	# of obtained spectra	MALDI good spectra (%)
*Anopheles* (Nys) *albimanus*	51	a–g	153	119	78
*Anopheles* (An) *apicimacula*	40	b, d, g	120	110	92
*Anopheles* (Nys) *aquasalis*	19	c, d	57	56	98
*Anopheles* (Nys) *darlingi*	14	b, g	42	40	95
*Anopheles* (An) *malefactor*	13	b, d, g	39	39	100
*Anopheles* (Nys) *nuneztovari*	66	b, g	198	192	97
*Anopheles* (An) *pseudopunctipennis*	15	b, g	45	45	100
*Anopheles* (An) *punctimacula*	32	b, d, g	96	81	84
*Anopheles* (Nys) *strodei*	16	e	48	48	100
*Anopheles* (Nys) *triannulatus*	9	a, f	27	26	96
*Anopheles* (Ker) *neivai*	10	c, f	30	24	80
*Chagasia bathana*	15	f	45	45	100
Total	300	7	900	825	92

(a) = Río Indio, Colón; (b) = Jaqué, Darién; (c) = Quebrada Hilo, Bocas del Toro; (d) = La Miel, Puerto Obaldía, Darién; (e) = Finca 51, Guabito, Changuinola, Bocas del Toro; (f) = Achiote, Colón; (g) = El Coco, Darién. Nys = Nyssorynchus; An = Anopheles; Ker = Kertezia. More information about these localities can be obtained from figures and maps in references [4, 6, 7]

### MALDI mass spectrometry parameters

The mass spectrometer used for the measurements was an UltrafleXtreme III (Bruker Daltonics, Bremen, Germany) equipped with a MALDI source, a time-of-flight (TOF) mass analyzer, and a 2 KHhz Smartbeam™-II neodymium-doped yttrium aluminum garnet (Nd:YAG) solid-state laser (λ = 355 nm) used in positive polarization mode. All spectra were acquired with an automatized script in the range of 2000 to 20,000 m/z in linear mode for the detection of the most abundant proteins. Every spectrum represents the accumulation of 5000 shots with 300 shots taken at a time, and the acquisition was done in random-walk mode with a laser power in the range of 50 to 100% (laser attenuation at 20%). To promote the accuracy of the identification algorithms, the spectra collected with the automatic script had to include at least one peak with a minimum intensity of 3500 arbitrary units [a.u] as a stringent parameter of quality to be considered “good quality” spectra. The software FlexAnalysis™ (Bruker) was used to analyse the spectra initially and to evaluate number of peaks, peak intensity and perform simple spectra comparisons to visually inspect for differences in dominant peaks that would suggest possible classification into discrete taxa. All samples were placed and measured on three individual target wells with spectra from three technical replicates collected per well.

### Data analysis, statistics and clustering algorithms

For routine mass spectra statistical analysis, including two-dimensional (2D) peak distributions and principal component analysis (PCA), the program ClintProTools™ (Bruker) was used. Individual sample spectra were pre-processed using smoothing and baseline subtraction functions, and three-dimensional (3D) plots were generated to display unsupervised clustering at the subgenera and species levels based on the most abundant protein spectra. However, complete classification of spectra from the field-collected mosquitoes could not be achieved with the manufacturer’s software because reference library entries that conformed to the quality standards of the application could not be created.

For more stringent and comprehensive data clustering and identification, a custom-made Linear Discriminant Analysis (LDA) quantitative approach was implemented using the software MATLAB^®^ (MathWorks, Natick, MA, USA). Given the size of the samples, a dimensionality reduction stage was implemented using PCA as well. Both approaches have been used in identification in the context of face recognition [27, 28], and are established methods used in spectral classification in the context of mass spectrometry [29, 30].

Let the training set of the samples be Γ_1_, Γ_2_, Γ_3_, …, Γ_*M*−1_, Γ_*M*_. The average sample is defined as $\Psi = \frac{1}{M}\sum_{i = 1}^{M} \Gamma_{i}$. Each sample differs from the average sample by the vector Φ_*i*_ = Γ_*i*_ − Ψ. Given the mean-centered sample matrix $A = \left[ {\Phi_{ 1} ,\Phi_{ 2} ,\Phi_{ 3} , \ldots ,\Phi_{M - 1} ,\Phi_{M} } \right]$, the covariance matrix $C = \frac{1}{M}\sum _{n = 1}^{M} \Phi_{n} \Phi^{T} = AA^{T}$ was calculated. The eigenvectors of this covariance matrix correspond to a set of orthonormal vectors that form a basis to represent the data with a reduced dimensionality. A previously published approach [28] was used to calculate indirectly the first M eigenvectors of the matrix C, by estimating the eigenvectors of the matrix *L* = *A*^*T*^*A*, reducing the memory and computational requirements of this procedure.

PCA-based identification consists in using the projection of the sample in the eigenvectors to calculate a set of coefficients $$ \begin{array}{*{20}c} {\omega_{k} = u_{k}^{T} \left[ {\Gamma -\Psi} \right],} & {k = 1,2,3, \ldots ,M' < M} \\ \end{array} $$ to describe each sample as a vector $\Omega^{T} = \left[ {\omega_{ 1} ,\omega_{ 2} , \ldots ,\omega_{{M^{\prime}}} } \right].$ The average of the vectors describing the samples of the training set of a given class was used to represent the class in the new basis. Then, to identify a test sample, the Euclidean distance between the vector Ω describing the test sample and the vectors describing each class were calculated. The class with the minimum distance with respect the test sample was assigned to the test sample. The PCA provides basis vectors that correspond to the direction of maximal variance in the sample space. In other words, using maximal variance as an unsupervised parameter for clustering, the test samples are then compared to the classes created with the information of mosquito species that were identified morphologically; if the distance between the test sample vector and the correct class (i.e., mosquito species) vector was the smallest one, this was considered a positive identification. In the other hand, LDA considers class information to provide a basis that best discriminates the classes [27]. The LDA can be applied over the data set expressed in terms of the coefficients obtained by the PCA. Thus, PCA reduces the dimensionality of the data, and the LDA provides supervised classification.

The LDA basis vectors $W_{opt} = \left[ {w_{1}  w_{2}  w_{3} \ldots  w_{P} } \right]$ are obtained by calculating the matrix that maximizes the ratio $\frac{{|W^{T} S_{B} W|}}{{|W^{T} S_{W} W|}}$, where *S*_*B*_ and *S*_*W*_ are the between-class scatter matrix and the within-class scatter matrix, respectively. This new set of vectors maximizes the distance between class means and minimizes the class variation. For test sample identification, a similar Euclidean distance approach was implemented, as explained for the PCA case. Thus, in this case using the between- and within-class scatter ratio vectors as supervised parameters for clustering, the test samples are compared to the LDA basis vectors that contain the information of mosquito species that were identified morphologically; if the distance between the test sample vector and the correct class (i.e., mosquito species) vector was the smallest one, this was considered a positive identification. The performance of the LDA approach was tested using Monte Carlo cross validation over 500 iterations. For each iteration, the data is split randomly in 80% of the samples for training and 20% of samples for testing, for each species. For such implementation, the first 50 vectors or components from the PCA stage were used, which after being projected for the LDA algorithm, also generated a 50 components data set. This number of components was chosen after a performance analysis using a Monte Carlo approach. This number provided the best identification rates. The total data set consists in 826 spectral samples of 12 species.

## Results

### Sample preparation and optimization

The three protein extraction protocols used herein were relatively simple and, in general, involved a combination of formic acid and organic solvent to solubilize the proteins present in each sample and facilitate their ionization (Table 1). Protocol #2 was the most time-consuming because of one washing step with water and 1-h period to incubate the sample. Protocol #1 was slightly faster than Protocol #2 since it did not involve the incubation of the sample; however, it was the most labor intensive due to one additional washing step with ethanol plus additional decantation and centrifuging steps. Protocol #3 involved only two steps, was the fastest and least labor intensive of the three protocols (Table 1). The two rinsing steps of Protocol #1 seemed to help reduce noise in the spectra and improve repeatability of each spectrum. A critical step in extraction protocol #1 was the homogenization of the samples. Physical homogenization with a manual stainless-steel pestle tool and a routing movement technique was used. Here the applied force and time of homogenization were important to obtain good quality spectra, probably due to the reduced size and weight of the samples (e.g., one leg). Protocol #1 provided robust results during preliminary examinations despite being the most labor intensive; thus, the influence of different body sections and the sex of mosquitoes in the mass spectra was analysed with protocol #1 only.

The different body sections of the mosquito presented specific and repeatable mass spectra regardless of species being analysed. In general, and across mosquito species the head and the thorax were the most signal-rich parts analysed. However, the legs, divided into anterior, middle and posterior pairs, showed more robust and repeatable signals (Additional file 3). Moreover, similar results were found for *A. albimanus*, *A. aegypti* and *A. albopictus* mosquitoes when using only one leg, so this sample preparation was chosen to minimize the amount of voucher used. Another question being evaluated was if differences in the sex of the mosquito could affect the spectra from the legs. The legs of male and female specimens of *A. albimanus*, *A. aegypti*, *A. albopictus* were compared and no evident differences were found in their spectra due to sex (Additional file 4). Given the repeatability and lower risk of spectra variations with the legs, and the presence of three pairs of legs per individual that could serve as technical replicates for future experiments, all further evaluations with field-collected samples of *Anopheles* were performed using female mosquito legs only.

### MALDI mass spectrometry to classify field-collected *Anopheles* species

**Fig. 1:**
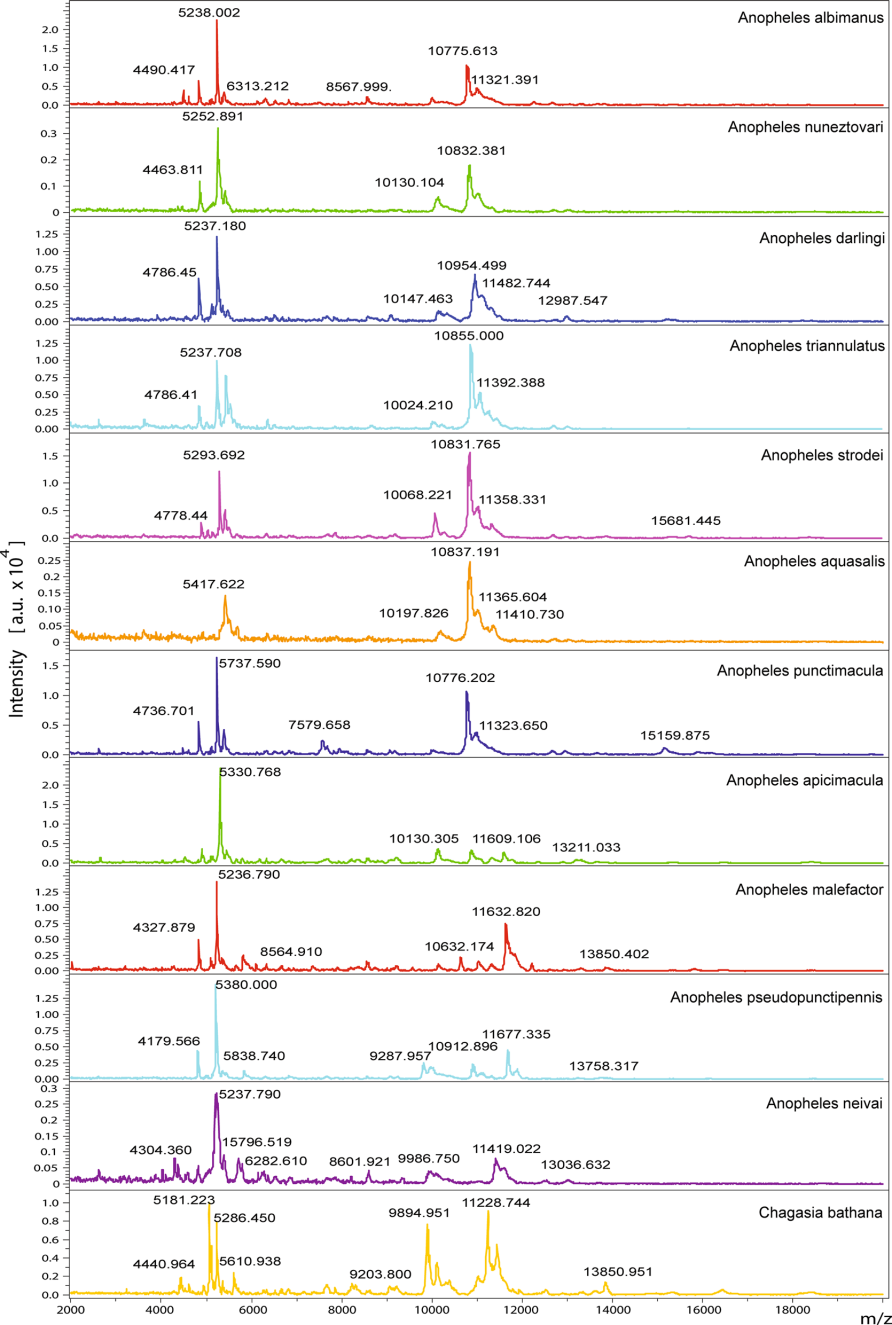
Baseline corrected and smoothed spectra for 11 species of mosquitoes in the genus *Anopheles* plus *Chagasia bathana*. Mayor peaks and their molecular weights are annotated in the range of 2000 to 20,000 m/z for all species

The mass spectra of field-collected *Anopheles* mosquitoes, in general, had lower quality than that of laboratory-reared mosquitoes in terms of intensity of the signal and signal-to-noise ratio, possibly due to contaminants that were not removed during the rinsing steps of the extraction protocol, which could have suppressed the ionization of certain molecules or introduced noise in the spectra (Table 2). The percentage of good quality spectra acquired from the prepared specimens in automatic mode with the MALDI mass spectrometer ranged from 78% for *A. albimanus* to 100% for several of the species, including *Anopheles malefactor*, *Anopheles pseudopunctipennis*, *Anopheles strodei* and *Chagasia bathana*. All biological specimens of *Anopheles* mosquitoes evaluated in this study were capable of generating good quality spectra (Table 2) and the specimens within each species showed consistently similar protein profiles after analysis with the MALDI technique, regardless of their taxonomic subgenera, collection date and/or sampling location. Mean protein spectra for *Anopheles* species differed visually among taxa and the differences appeared to be related to their phylogenetic relationships (Fig. 1). For example, species within the subgenus Nyssorynchus of Anopheles were more similar among them in terms of pick number and position, than with other taxa from a different subgenus. Nonetheless, somehow seemingly closely related species, such as *Anopheles punctimacula* and *Anopheles malefactor* within the Arribalzagia Series of the subgenus Anopheles, depicted reasonably distinct protein spectra that motivated the pursue of clustering algorithms for their identification (Fig. 1).

**Fig. 2:**
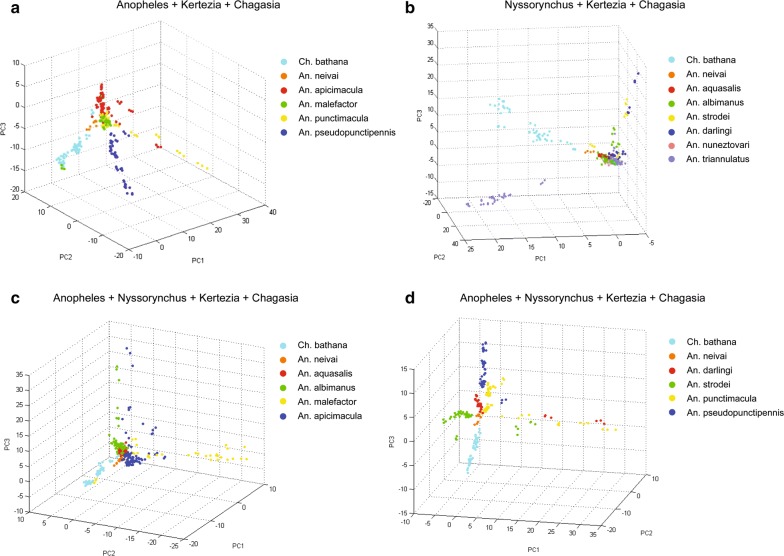
Principal component analysis (PCA) of individual observations plotted against first, second and third principal components (PC). **a** All species belonging to the Anopheles subgenus of *Anopheles*, including three of them belonging to the Series Arribalzagia within this same subgenus as well (i.e., *Anopheles apicimacula*, *A. malefactor* and *Anopheles punctimacula*), were clustered in comparison to the *Kertezia* and *Chagasia* subgenera. **b** A similar analysis was performed for the *Nyssorynchus* subgenus compared to the same *Kertezia* & *Chagasia* species and **c**, **d** with all four subgenera together in the same analysis, picking only two species of each of the subgenera that had more abundant species. Different colors represent different species

Distinct mass spectra profiles between morphologically-identified *Anopheles* species could be classified by an unsupervised PCA algorithm to identify specimens. The quantitative performance of the PCA algorithm was assessed per species (Table 3), and visually confirmed with the clustering exhibited in 3D plots (Fig. 2). The PCA global positive identification rate was 89.83%, with 7 out of 12 species having higher than 90% positive identification rate. For visualization purposes in the PCA scores plots, all species that were morphologically identified within the Anopheles or Nyssorynchus subgenera were separately compared against Kertezia and Chagasia, for which there was only one species in each. Three of the species in the Anopheles subgenus belonged to the Arribalzagia Series within this same subgenus as well (i.e., *Anopheles apicimacula*, *A. malefactor* and *A. punctimacula*). The PCA 3D graph showed that each species separated in well-defined clusters, and the distance among clusters seemed to be related to the phylogenetic relationships as evidenced by the clear separation from the specimens of *Chagasia bathana* (Fig. 2a, b). All the subgenera together were also compared using only two species from each Anopheles and Nyssorynchus subgenera, for visualization purposes. Again, the spectra from specimens of each species clearly clustered together, with reasonable overlap between groups (Fig. 2c, d).

**Table 3:** Performance of PCA and LDA clustering algorithms

Species name	PCA positive identification rate (%)	LDA positive identification rate (%)	Spectra per class	# Training elements	# Test elements
*Anopheles albimanus*	91.48	97.40	119	95,000	24,000
*Anopheles apicimacula*	94.54	96.30	110	88,000	22,000
*Anopheles aquasalis*	99.99	99.90	56	44,000	12,000
*Anopheles darlingi*	95.65	99.30	40	32,000	8000
*Anopheles malefactor*	71.81	67.88	39	31,000	8000
*Anopheles nuneztovari*	81.47	86.73	192	153,000	39,000
*Anopheles pseudopunctipennis*	100.00	99.99	45	36,000	9000
*Anopheles punctimacula*	86.55	95.16	81	64,000	17,000
*Anopheles strodei*	88.75	93.63	48	39,000	10,000
*Anopheles triannulatus*	88.48	90.73	26	20,000	6000
*Anopheles neivai*	100.00	100.00	24	19,000	5000
*Chagasia bathana*	100.00	100.00	45	36,000	9000
Global	89.83	93.33	825	657,000	169,000

**Fig. 3:**
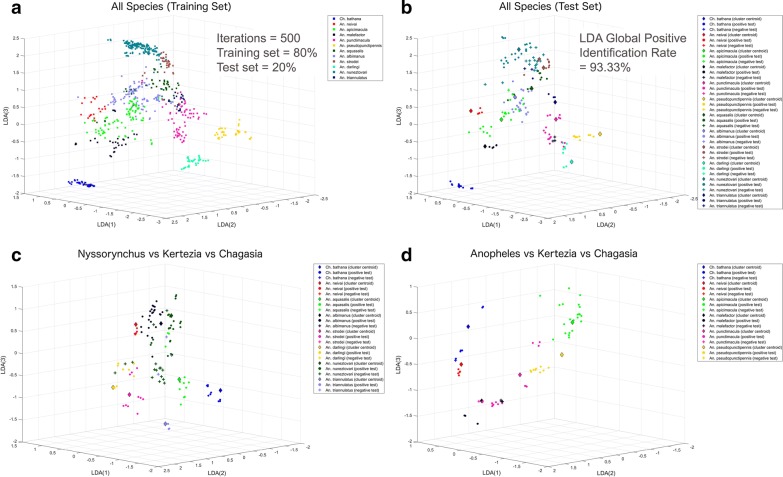
Linear discriminant analysis (LDA) applied to mosquito species of the subgenera Anopheles, Nyssorhynchus, *Anopheles* (Kertezia) *neivai* and *Chagasia bathana*. **a** Plot of the training set for all species projected over the first three components of the LDA. **b** Plot of the test set for all species projected over the first three components of the LDA. **c** Plot of the test set in the Nyssorynchus subgenera compared to the *Anopheles* (Kertezia) *neivai* and *Chagasia bathana* species, projected over the first three components of the LDA. **d** Plot of the test set in the Anopheles subgenera compared to the *Anopheles* (Kertezia) *neivai* and *Chagasia bathana* species projected over the first three components of the LDA. These 3D plots represent only one of the 500 Monte Carlo iterations performed with the algorithm. The algorithm had a 93.33% global positive identification rate

In addition, LDA analysis for all 12 species was performed using a Monte Carlo simulation with 500 iterations to optimize training and cross-validation prediction success rates (Fig. 3; Table 3). From all morphologically-identified species, a training set with 80% of the samples was randomly selected (Fig. 3a) and the other 20% was used as a test set (Fig. 3b), and this process was repeated in each iteration. Global and class positive identification rates were calculated to establish the classification capacity of the algorithm (Table 3). The positive identification rate corresponds to the percent ratio between positive identifications performed by the algorithm and the real positive cases in the data. The global positive identification rate obtained with the LDA was 93.33% (Table 3, Fig. 3b), with a range that went from 100% (best score possible) for *Anopheles neivai* and *Chagasia bathana*, to 67.88% for *A. malefactor*. For visualization purposes, the LDA representation of the *Nysorrynchus* (Fig. 3c) or *Anopheles* (Fig. 3d) subgenera compared against the subgenera Kertezia and Chagasia (one species each) were also plotted. The LDA clustering plots show that when comparing species from different subgenera and even within a particular subgenus, the separation between specimens from different species is evident.

## Discussion

### Addressing the limitations of previous studies with the MALDI

Proof of concept with the MALDI mass spectrometry to examine species boundaries among arthropod vectors of diseases has been well established before in ticks (Ixodidae—*Rhipicephalus*) [16, 18], fleas (Pulicidae—*Ctenocephalides*) [17], tsetse flies (*Glossina* spp.) [19], sandflies (Psychodidae—*Phlebotomus*) [21, 31], biting midges (Ceratopogonidae—*Culicoides*) [32] and mosquitoes (Culicidae) [10, 20, 22–25]. However, many of the experiments conducted up to now with the MALDI involved laboratory-reared specimens and few species or geographically discrete specimens of the same species. Also, with some recent exceptions [9, 10, 23–25], full arthropod bodies were largely used in their protocols, leaving no morphological vouchers for trial confirmation and replication. Moreover, some of these publications employed fairly distinct sample processing protocols, thus making it difficult to decide about their appropriateness and usefulness to study different arthropod groupings. Different methodologies to handle samples with the MALDI mass spectrometry might result in different outcomes, yet few published studies have evaluated the influence of these differences on the resulting protein spectra.

Here, a methodology was adjusted to use mosquitoes of the same sex (i.e., only females) that were processed for a specific body part (e.g., only legs) and with the best protein extraction protocol based on comparisons assumed on initial experiments using lab-colonized mosquitoes (i.e., Protocol #1). The MALDI mass spectrometry technique could also be used effectively and timely to discriminate among field-collected female individuals of various Neotropical *Anopheles* species using only one leg, while maintaining good signal robustness. The use of legs to generate protein spectra from ticks and mosquitoes with the MALDI has been successfully accomplished before [9, 10, 16, 23–25], yet so far this approach has not been used to classify samples of Neotropical *Anopheles* species, nor has it been applied to field collected specimens that were stored in silica gel.

Considering that one of the objectives of this study was to find the smallest portion of the mosquito that contained enough identifiable information in order to preserve the specimen voucher for other molecular eco-epidemiological assays, the results found with only one of the legs per specimen are very attractive due to the possibility of keeping almost the entire insect body to investigate phylogenetic relationship, pathogen infection rate, and identification of host blood type. Nevertheless, the intensity of the spectra collected with MALDI may be decreased when working with field-collected samples and such limited amount of biological material for homogenization. Still, in this study 92% of the analysed matrix-sample spots offered spectra with high-enough intensity to be picked up by the automatized script (e.g., 825 out of 897 spots from the three technical replicates per specimen), and only 3 of 12 tested species had a spectra collection rate below 90%. Since the groups with the lower spectra success rate included some of the more abundant species such as *A. albimanus* (78%), *A. punctimacula* (84%) and *A. neivai* (80%), and were equally likely across different localities and sampling dates, the lower spectra collection rate could potentially be due to degradation of some samples under unfavorable storage condition, failure to load samples successfully in the metal plate of the MALDI or contamination from the field. However, the procedure allows researchers to try again several times by using any of the remaining legs of the mosquito, thus offering a practical and realistic way around this problem. Future studies will have to test additional conservation methods and determine if preserving samples in silica gel was the cause of low success rates in obtaining the expected number of spectra overall and per species.

### A way around working without a reference library of protein spectra

The conventional MALDI biotyper approach for species identification uses a reference library database of laboratory-reared and well-characterized species-specific protein spectra plus computational software from the vendor to compare unknown spectra to those in the reference library. The program generates a degree of similarity between sample spectra and the reference library, and gives a simplified score ranging from 0.0 to 3.0, in which any score above or equal to 2.7, represents a perfect match between a sample spectrum and a particular library spectrum and 2.3 can be used as a minimum threshold for an accurate identification at the species level. This methodology has been very successful for clinical studies involving pathogenic bacteria to humans because they are easy to cultivate in the laboratory and their colony-forming units offer robust and repeatable signals [33]. However, to build a reference library with fresh and well-curated *Anopheles* species requires high-quality, extremely consistent spectra from mosquitoes collected in the field as immature stages and lab-reared in the insectary, which is complicated to accomplish either due to difficulties in field collecting larvae of some species or laboratory-rearing them in the insectary [34]. To date, only partial reference libraries with protein spectra from a mixture of laboratory-reared and field-collected mosquitoes have been built with mixed quality standards, forcing the use of alternating lower threshold scores for species identification of 1.8 [9, 10, 23–25] or as low as 1.3 in recent studies [35]. In addition, none of these studies have included Neotropical *Anopheles* species.

The quality of the spectra from the field-collected mosquitoes analysed in this study was lower than expected, requiring the use of other statistical techniques for identification. Mass fingerprinting for the identification of field-collected specimens that do not exist in a reference library or for those whose reference spectra cannot be generated, requires alternative approaches that can be developed to detect distinctive features in the spectra of unknown samples. To address this shortcoming, smoothed and baseline corrected spectra were produced from field-collected samples of 11 species of mosquitoes in the genus *Anopheles* plus *Chagasia bathana* and compared against the mean spectra from the same field samples as a self-curated reference library. Further, a combination of unsupervised (PCA) and supervised mathematical algorithms (LDA) were used to classify mass spectra of field-collected *Anopheles* with high consistency.

In general, PCA outcomes were less discriminant and robust than LDA, still PCA discriminated among *Anopheles* species from different subgenera with almost 90% accuracy and consistency. LDA was able to classify all 12 species of mosquitoes together with validation and cross-validation scores above 93%, both between and within subgenera. This included samples from seven localities across the entire country of Panama, including vectors and non-vectors of *Plasmodium*. Evidently, the clustering algorithm was more accurate for mosquito species that were phylogenetically distinct from the rest (i.e., *Kertezia* and *Chagasia* subgenera), with 100% success rate in these cases; while the success rate decreased for more closely related species (i.e., *A. malefactor*, from the Arribalzagia Series). Still, the global success rate was 93.33%, which is reasonably precise. Therefore, due to its supervised nature LDA was able to identify field-collected *Anopheles* species without the need of a reference library of species-specific protein spectra, and with higher resolution and discriminant power than PCA.

## Conclusion

A methodology was developed that allows the identification of field-collected mosquitos from the *Anopheles* genus without prior establishment of a reference library of well-curated lab-reared mosquitoes. Prior scientific work in Panama and elsewhere suggests that DNA barcodes occasionally fail to elucidate the evolutionary relationships among closely related *Anopheles* species. Although the number of mosquitoes analysed in this study is still relatively low, the results show that the classification algorithms used here were capable of clustering and identifying spectra from up to 12 different field-collected mosquito species. In future studies, this MALDI procedure will be tested to discriminate between geographically isolated populations/lineages of cryptic species complexes such as *A. punctimacula* sensu lato (s.l.) and *Anopheles apicimacula* s.l. This approach can be easily adapted and applied more broadly to other tropical regions of the world where *Anopheles* species diversity is high and morphological species complexes do exist.

## Additional files

**Additional file 1.** Optical micrographs of three mosquito species: (A, B and C) Images of lab-reared *Anopheles albimanus* (A), *Aedes aegypti* (B) and *Aedes albopictus* (C) (Left to right in that order) used for the optimization of the sample preparation protocol and MALDI mass spectrometry analysis.

**Additional file 2.** Magnified images of different body parts of *Aedes albopictus* used to generate protein spectra with the MALDI mass spectrometry approach.

**Additional file 3.** Protein spectra generated from different body parts of female mosquitoes (On the left side). Optical micrographs of the head, thorax, abdomen, wings and anterior, middle and posterior legs of laboratory-reared *Aedes albopictus* (On the right side, in that order).

**Additional file 4.** Comparison of protein spectra generated from the middle legs of males (Top) and females (Bottom) of *Anopheles albimanus*, *Aedes aegypti* and *Aedes albopictus* mosquitoes.

**Additional file 5.** Mosquito trapping methods: (A) Intersection trap; (B) Shannon trap; (C) CDC Miniature Light trap; (D) Larvae collection and (E) sample processing procedure used in the study.

## Abbreviations

MALDI: matrix-assisted laser desorption/ionization
PCA: principal component analysis
LDA: linear discriminant analysis
DNA: deoxyribonucleic acid
INDICASAT: Institute for Scientific Research and High Technology Services
STRI: Smithsonian Tropical Research Institute
SNI: National System of Investigation
MHIRT: Minority Health and Health Disparities International Research Training Program
NIH: National Institute of Health
UTP: Technological University of Panama
TOF: time-of-flight
MINSA: Ministry of Health
MiAmbiente: Ministry of Environment
SENAFRONT: National Border Protection Service
CDC: Center for Disease Control
