# Supplementary figures and images for: Application of matrix-assisted laser desorption/ionization mass spectrometry to identify species of Neotropical Anopheles vectors of malaria
Source: Malar J. 2019 Mar 22;18:95. doi: 10.1186/s12936-019-2723-0 (PMC6431007; doi:10.1186/s12936-019-2723-0)

## Slide 1
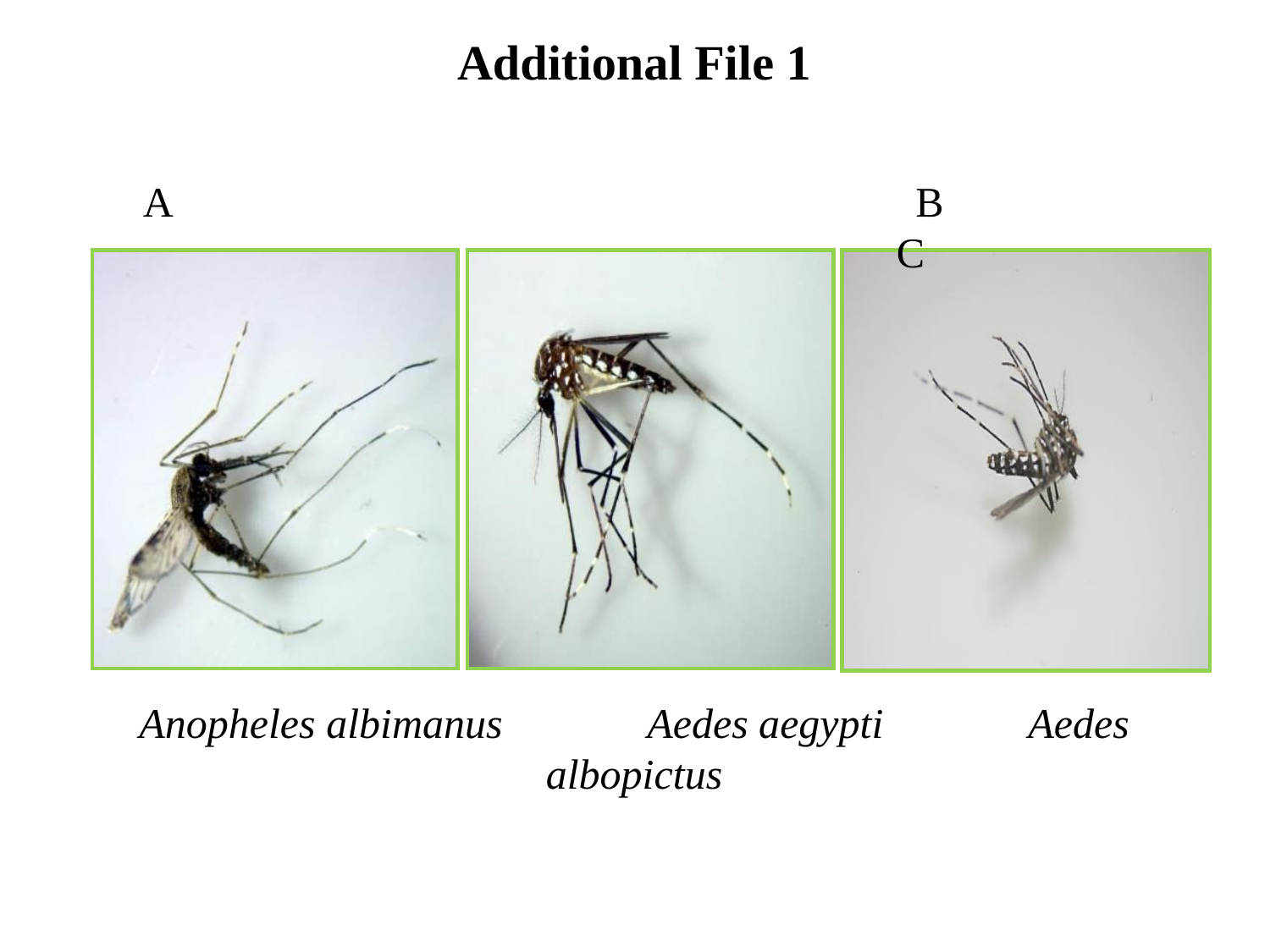

Additional File 1
A						 B						 C
Anopheles albimanus	 	Aedes aegypti	 	Aedes albopictus

Supplement: Supplementary file 1 — Additional file 1. Optical micrographs of three mosquito species: (A, B and C) Images of lab-reared Anopheles albimanus (A), Aedes aegypti (B) and Aedes albopictus (C) (Left to right in that order) used for the optimization of the sample preparation protocol and MALDI mass spectrometry analysis. [file 12936_2019_2723_MOESM1_ESM.pptx]

## Slide 1
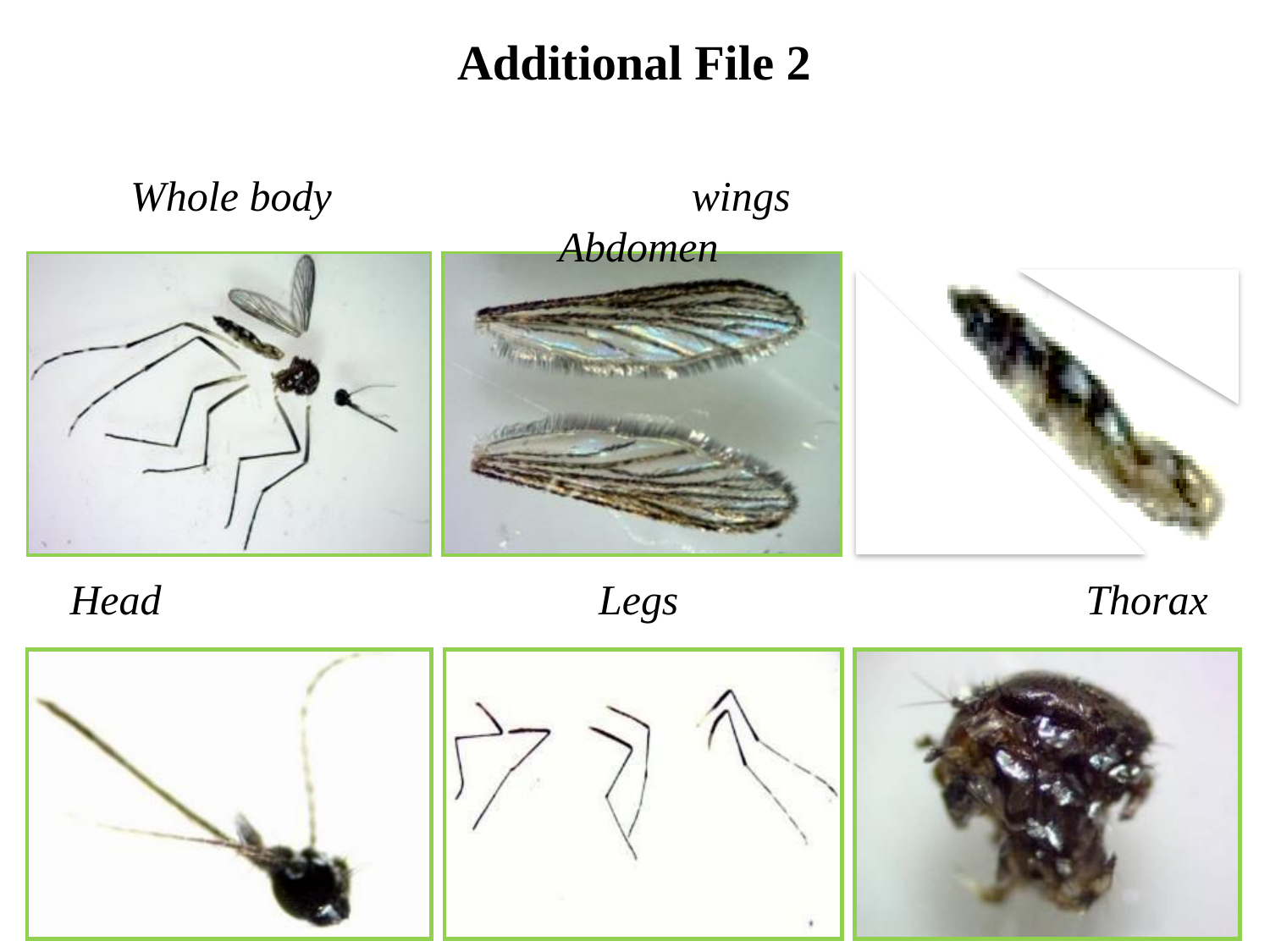

Additional File 2
Whole body	 	 wings 	 	Abdomen
Head	 	 Legs 	 	Thorax

Supplement: Supplementary file 2 — Additional file 2. Magnified images of different body parts of Aedes albopictus used to generate protein spectra with the MALDI mass spectrometry approach. [file 12936_2019_2723_MOESM2_ESM.pptx]

## Slide 1
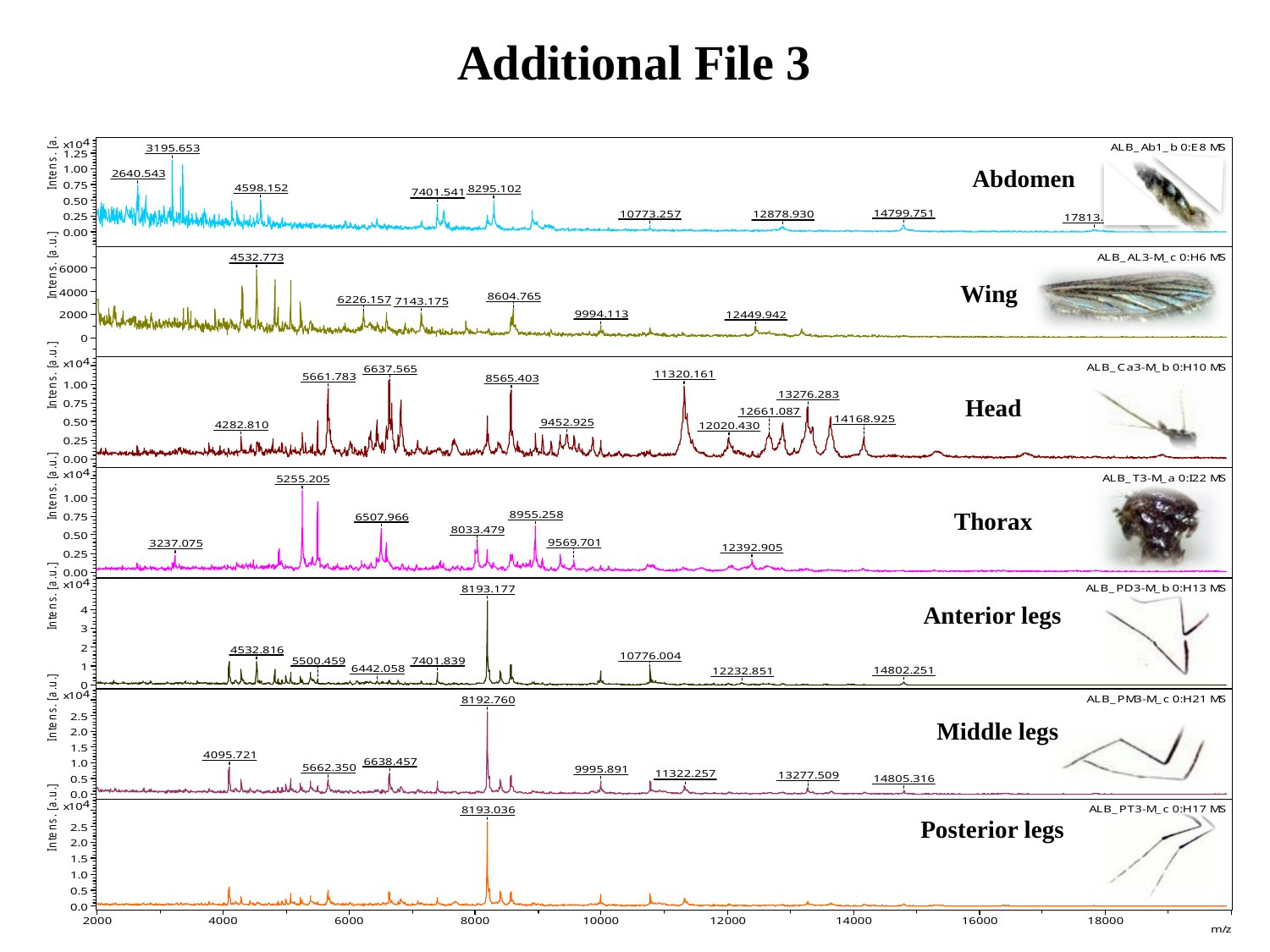

Additional File 3
Abdomen
Wing
Head
Thorax
Anterior legs
Middle legs
Posterior legs

Supplement: Supplementary file 3 — Additional file 3. Protein spectra generated from different body parts of female mosquitoes (On the left side). Optical micrographs of the head, thorax, abdomen, wings and anterior, middle and posterior legs of laboratory-reared Aedes albopictus (On the right side, in that order). [file 12936_2019_2723_MOESM3_ESM.pptx]

## Slide 1
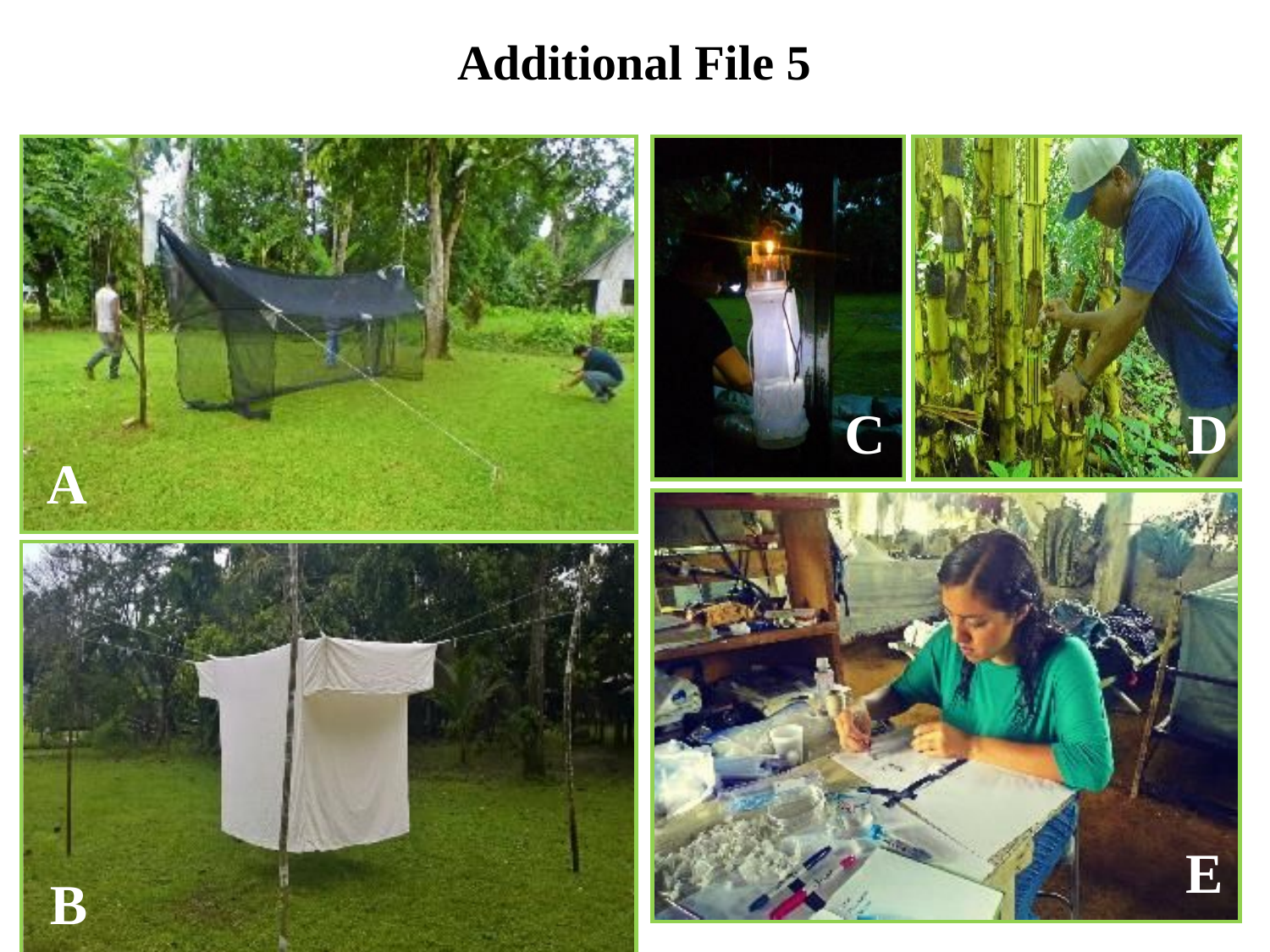

Additional File 5
C
D
A
E
B

Supplement: Supplementary file 5 — Additional file 5. Mosquito trapping methods: (A) Intersection trap; (B) Shannon trap; (C) CDC Miniature Light trap; (D) Larvae collection and (E) sample processing procedure used in the study. [file 12936_2019_2723_MOESM5_ESM.pptx]
