# Supplementary material for: Application of matrix-assisted laser desorption/ionization mass spectrometry to identify species of Neotropical Anopheles vectors of malaria
Source: Malar J. 2019 Mar 22;18:95. doi: 10.1186/s12936-019-2723-0 (PMC6431007; doi:10.1186/s12936-019-2723-0)
Supplement: Supplementary file 4 — Additional file 4. Comparison of protein spectra generated from the middle legs of males (Top) and females (Bottom) of Anopheles albimanus, Aedes aegypti and Aedes albopictus mosquitoes. [file 12936_2019_2723_MOESM4_ESM.pptx]

## Slide 1
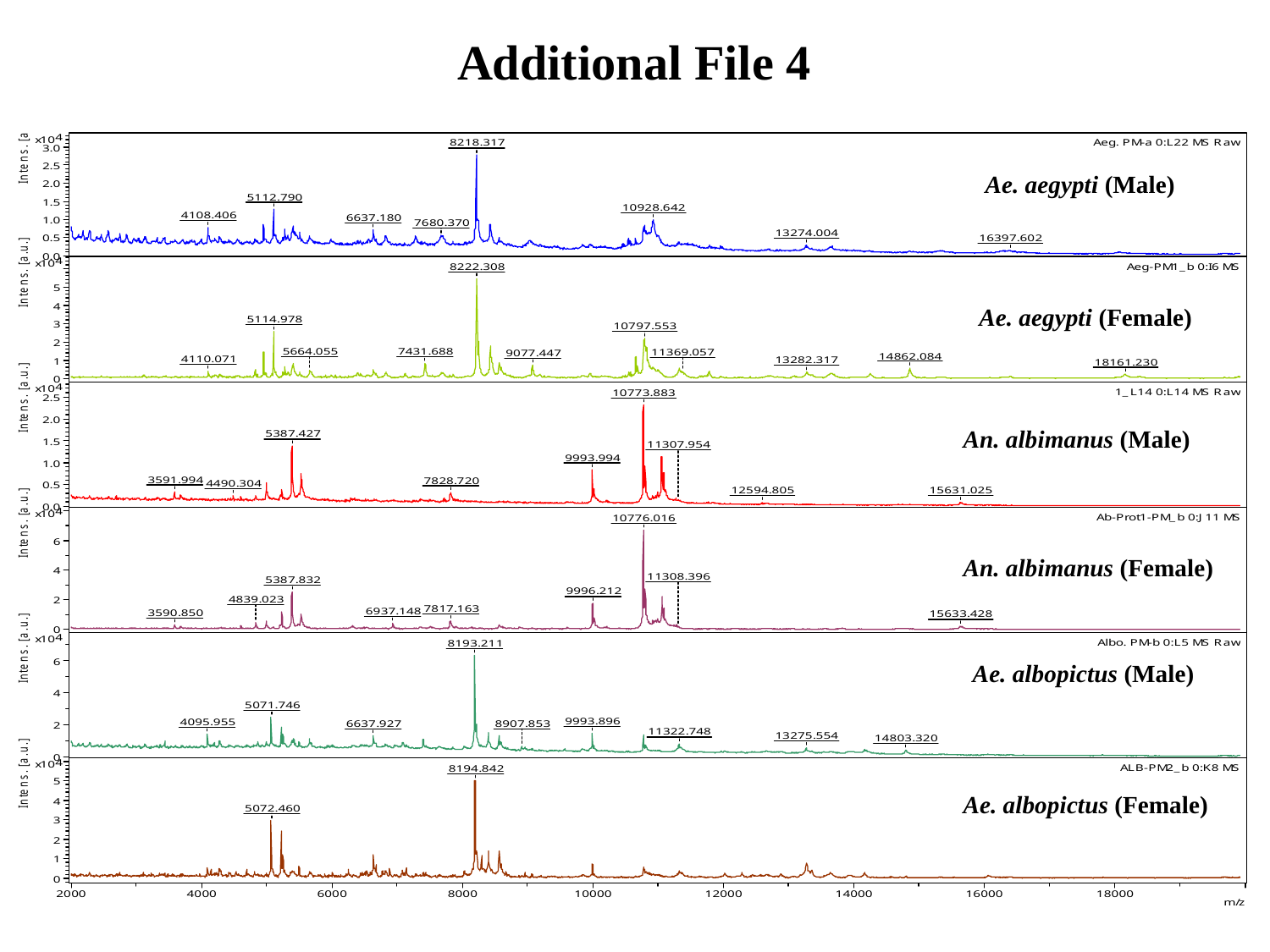

Additional File 4
Ae. aegypti (Male)
Ae. aegypti (Female)
An. albimanus (Male)
An. albimanus (Female)
Ae. albopictus (Male)
Ae. albopictus (Female)
